# Reduced holey graphene oxide film and carbon nanotubes sandwich structure as a binder-free electrode material for supercapcitor

**DOI:** 10.1038/s41598-020-58162-9

**Published:** 2020-02-11

**Authors:** Khan Abdul Sammed, Lujun Pan, Muhammad Asif, Muhammad Usman, Tianze Cong, Farid Amjad, Muhammad Asif Imran

**Affiliations:** 1 School of Physics, Dalian University of Technology, Dalian, 116024 PR of China; 2 Department of Materials Science and Engineering College of Engineering, Peking University, Beijing, 100871 China; 3 School of Chemical Engineering, Dalian University of Technology, Dalian, 116024 PR of China; 4 Department of Physics, Khwaja Fareed University of Engg. and Information Technology, Rahimyar, Khan-64200 Pakistan

**Keywords:** Materials for devices, Materials for energy and catalysis

## Abstract

A novel carbon nanotubes (CNTs) and reduced holey graphene oxide film (RHGOF) sandwich structure has been fabricated to enhance its electrochemical properties. CNTs are grown by a catalyst assisted chemical vapor deposition technique, interpenetrated between the RHGOF layers. A RHGOF/CNTs hybrid film is used as a binder-free supercapacitor electrode. The grown CNTs in the graphene layers structure act as spacers and bridges to increase the counductivity of RHGOF, while the grown CNTs on the surfaces of the graphene contribute to increase the specific surface area of RHGOF. The results demonstrate that the synthesized porous, flexible and binder free hybrid electrode has advantages of higher ion diffusion rate, longer diffusion length and larger ion accessible surface area as compared to the pristine graphene which results in an extra ordinary galvanostatic charge-discharge specific capacitance of 557 F/g at a current density of 0.5 A/g, with excellent rate capabilities and superior cyclic stabilities.

## Introduction

In recent era, environment adaptability, portability and compatibility of electronics, hybrid electric vehicles are becoming challenging issues. Energy storage devices having high performance are the part and parcel of these aforementioned devices^1^. Because of its promising adventages, supercapacitor is one of the fascinating energy storage device, such as fast dynamic response, high power density, longer life span and good capability rate^2,3^.Therefore, explicit studies of the characteristics of supercapcitor electrode materials are very essential components that ultimately affect its electrochemical properties^1,4–7^. Most recently, researchers are trying their best to prepare flexible type of electrode materials possessing good cyclic stability, high performance output in terms of charge-discharge rates and specific capacitance.

Graphene (GR), being a 2D nano-structured material, has already been acknowledged as an attractive material for supercapacitor electrode. As a single thick sheet of carbon atoms, due to the numerous profound physic-chemical properties,it has been presaged for transfiguring a wide range of technological area^8–11^. Moreover, it has high unique mechanical flexibility intrinsic electrical conductivity, theoretical gravimetric capacitance of about 550 F/g and remarkably large theoretical surface area of 2630 m^2^/g^11–13^. On the contrary, due to the extended π-conjugation in the basal plane, pristine GR has van der Waals forces and π-π interaction. Thus, GR sheets are stacked with each other under these effects. Their irreversible agglomeration not only hinders the ion diffusion rate, but also decreases accessible surface area and consequent reduction in the gravimetric capacitance^14–17^. To improve its capacitive performance, numerous methodologies have already been proposed. For instance, to prevent the restacking, conducting carbon materials as an active channel (such as carbon black, carbon nanotubes (CNTs)) and conducting polymers (such as polyaniline), have been used. Additionally, nanostructured pseudo capacitive materials (such as MnO_2_, and Mn_3_O_4_ etc.) were also used to enhance capacitive properties^18–23^.

Recently, CNT/GR hybrid materials have been realized as the most capable candidate for electrode materials because of their improved capacitive properties and high flexibility. Many studies on CNT/GR hybrids are reported in literature. For instance, Yan *et al*. synthesized CNT/GR hybrid materials on nickel foam by using a two-step chemical vapor deposition (CVD) method and reported 104 F/g specific capacitance in their composite^24^. Moreover Fan *et al*. produced CNT/GR hybrid composite by the arrangement of microwave methods and CVD technique that shown high specific capacitance of 385 F/g at 10 mV scan rate^25^. However mostly GR/CNT hybrid electrode composites were synthesized by using binders because they were in the form of powder. Mean while, Xiong *et al*. adopted the combination of floating catalyst CVD and electrophoretic deposition that led to the preparation of GR/CNT/nickel foam hybrid materials, having promising 236.18 F/g specific capacitance. Although this composite can be used directly as supercapacitor electrode^26^, the GR/CNT cannot be used independently without the support of Ni foam. Therefore, currently free-standing flexible CNT/GR composite with adequate capacitive properties is the need of hour to extend its applications. For free standing, Fan *et al*. used polystyrene colloidal particles as template and synthesized 3D porous nitrogen doped GR/CNT flexible paper to enhance capacitive properties^27^. They reported specific capacitance of 294 F/g at 1 A/g current density in 6 M KOH aqueous electrolyte.

Even though the already published GR/CNT film electrodes have shown good capacitive properties, still these characteristics are limited because of the π-π stacking of GR. Hence synthesizing holey graphene (HGR) is a rational idea to remove π-π stacking and produce good capability rates and shorten the ion diffusion path^28–31^. For instance, Yu *et al*. prepared HGR oxide and its derivative macrostructures which exhibited significantly higher accessible surface area as well as ion diffusion rate as compared to non-holey composites^28^. These binder free porous composites were used directly as supercapacitor components, which displayed the specific capacitance of 283 F/g. Cheng *et al*. prepared flexible and porous HGR paper by using vacuum filtration technique. This paper was used directly as supercapacitor electrodes, exhibiting a specific capacitance of 201 F/g at 1 A/g with excellent cyclic stabilities and good rate capabilities^32^. Deng *et al*. prepared a CNT/HGR flexible free standing film by using vacuum filtration of the mixed functional CNTs and HGR solutions, the prepared CNT/HGR hybrid electrode material exhibited an ultra-high specific capacitance of 268 F/g at 0.25 A/g with superior cyclic stabilities and excellent rate capabilities. Although their composite showed a good output but CNTs are agglomerated during the solution based approach. Hence, whole area of HGR as substrate cannot be utilized^33^.

To address the aforementioned concerns, a facile method is devised to synthesize CNTs and reduced holey graphene oxide film (RHGOF) hybrid multi-layered sandwich structure as a binder-free electrode by the combination of vacuum filtration technique and CVD. This 3D structure is not only supportive to accelerate electron transfer during charge-discharge process but also beneficial in utilizing specific surface area to a greater extent.

## Characterization

### Electrochemical characterizations

Electrochemical performances of the samples were analyzed by Galvanostatic charge-discharge (CD), cyclic voltammetry (CV), and electrochemical impedance spectroscopy (EIS). Electrochemical work station (CHI660E instruments Inc. China) was used to carry out aforementioned characterization by using three electrode system, which includes Hg/HgO as a reference electrode, a piece of Pt as counter electrode, and as prepared binder free flexible electrode (RHGOF/CNTs) as working electrode in a 6 M KOH electrolyte solution. This system was used to measure CV and CD at scan rates of 5 to 100 mV/s under a potential window from −0.9 to −0.1 V and at different current densities from 0.5 to 6 A/g, respectively. The specific capacitance of electrodes was calculated from CD curves through the following formula.

$${\rm{C}}=\frac{{\rm{It}}}{\Delta {\rm{V}}.{\rm{m}}}$$

where constant discharge current (A) is represented by I, mass of active material (g) by m and the t shows the time of discharge stage(s) and ΔV is the potential difference of charging(V). Time relaxation constant was measured by the following equation.

$${{t}}_{o}=\frac{1}{{{f}}_{o}}$$

whereas, *f*_0_ represents characteristic frequency and t_0_ is time relaxation constant. At a phase angle of −45°, t_0_ stands for the minimum time needed to discharge all the energy from the device with an efficiency of more than 50%.

## Results and Discussion

**Figure 1:**
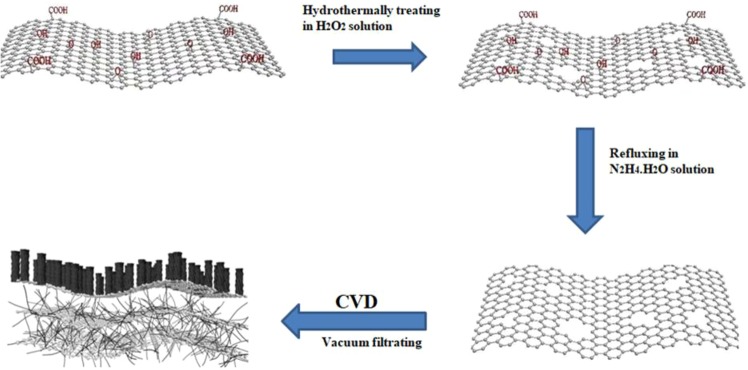
Formation scheme of RHGOF/CNTs sandwich structure.

The formation scheme of binder-free sandwich structure containing RHGOF and CNTs is illustrated in Fig. 1. The GO was etched by hydrothermal reaction with H_2_O_2_ at low temperature and was reduced with hydrazine, respectively. Final suspension was filtered out in order to get flexible RHGO film. Later on, RHGOF/CNTs sandwich structure was obtained after applying CVD method.

Actually, GO nano sheets possess numerous active sites. These sites were further etched to generate porous carbon vacancies on the surface. The porous structure of the sample was confirmed by nitrogen desorption/adsorption analysis. Nitrogen adsorption/desorption tests showed the RHGOF exhibited a much higher Brunauer−Emmett−Teller specific surface area of ∼560.8 m^2^/g than RGOF (∼254.6 m^2^/g) and a Barrett−Joyner−Halenda average pore size distribution is 3.25 nm. (Surface area data are shown in the Supporting Information). It is observed that the surface area of graphene within the RHGOF is highly accessible due to the nanopores in the basal plan inspite of the compact stacking structure. Particularly, the nanopores in RHGOF not only promoted ion diffusion and access to the graphene surface than in RGOF nanosheets^28^, it also provides a short pathways for electrolyte to penetrate inside the layers that directly enhance the accessible surface area.

Moreover due to the sandwich structure of RHGOF/CNTs composite, CNTs acting as spacer and provides conducting bridges in RHGOF/CNTs. Hence larger accessible surface areas are offered. More electron and ion transfer pathways are generated in favor of the electrolyte penetration. The obtained flexural strength (ϭ) and modulus of elasticity(E) of RHGOF/CNTs are 4.4 TPa and 5.21 TPa respectively. These values are significantly higher than published^34^.

**Figure 2:**
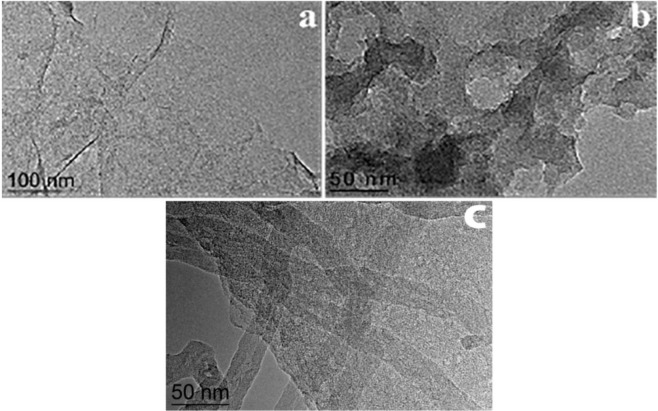
(**a**) TEM image RGOF **(b)** TEM image of RHGOF, **(c)** TEM image of RHGOF/CNTs hybrid composite.

Figure 2a, TEM image of RGOF that reveals very stable nature of transparent sheets.

While, RHGOF analysis is evidenced as non-transparent sheets morphology mainly due to the efficient etching of GO nanosheets by H_2_O_2_. Moreover, it also exhibited that across the basel plane of 2D HGO, there is enriched in-plane pores with ranging few nanometers size (Fig. 2b). In Fig. 2c TEM of RHGOF/CNTs clearly shows that surface of HGR sheet was combined with CNTs significantly, having an average diameter of 24 nm. Additionally, Fig. 2c reveals that in flexible films CNTs serve as a physical spacer. Hence, a precise porous sandwich structure is formed, which provided enough pathways to electrolyte between the HGR sheets. This sandwich structure RHGOF/CNTs is considered to be a more promising super-electrochemical capacitor (SEC) electrode material as compared to RGOF and RHGOF. Because interlayer space between the nanosheets of RGOF and RHGOF was too small to penetrate the electrolyte as compared to RHGOF/CNTs^35–37^.

**Figure 3:**
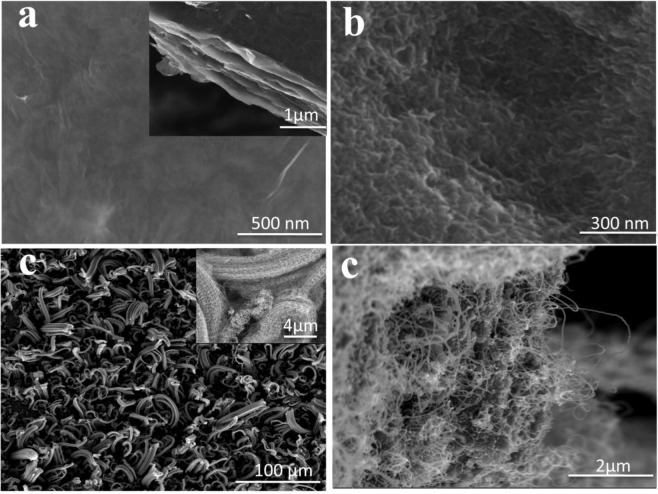
(**a**) FESEM images of RGOF, cross-sectional image of RGOF inset of (**a**,**b**) RHGOF, (**c**) RHGOF/CNTs hybrid composite, magnified image of RHGOF/CNTsis inset of (**c**,**d**) Cross-sectional image of RHGOF/CNTs.

FESEM images of prepared samples (RGOF, RHGOF and RHGOF/CNTs) are shown in Fig. 3. Figure 3a shows RGOF no flakes on the surface, rather reveal grooves. These fluctuations might be caused by overlaping of GO nanosheets causing aggregations during the filtration process.

The cross-section of RGOF paper elucidating layered structure that was also reported in the published research^27^ (Fig. 3a inset). On the other hand, FESEM of RHGOF shows porous and flake-like structures. During filtration process flake-like graphene nanosheets are assembled that formed a flexible and compact layered structures with larger specific surface area as compared to RGOF (Fig. 3b). In this work, CNTs were grown through the CVD method. Figure 3c depicts bundles of CNTs on the upper surface of the RHGOF. Moreover, cross-section of hybrid RHGOF/CNTs reveals that strings of CNTs were grown inside the layers (Fig. 3d). This is due to exposure of large surface area to the C_2_H_2_ and Ar to do the promising reaction at upper surface of the film. Preceding more it is found that, bundles were uniformly distributed along the surface. Inset of Fig. 3c shows high resolution image of CNTs bundles at the upper surface of the RHGOF film.

**Figure 4:**
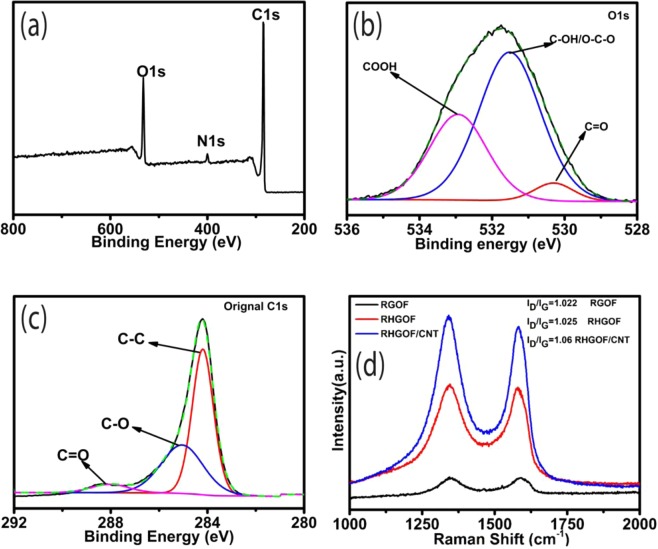
(**a**) XPS survey, (**b**) O1s spectra, and (**c**) C1s spectra for RHGOF. (**d**) Raman of RGOF, RHGOF and RHGOF/CNTs.

The chemical states and binding energies were identified and analyzed by XPS, as shown in Fig. 4a. It is confirmed from the survey image that the C and O are the main elements in the prepared RHGOF sample along with small percentage of N atoms. Figure 4c reveals that the C1s spectrum of RHGOF is constituted into three sub-peaks. These peaks centered at 288.05, 285.05 and 284.2 eV corresponding to C=O, C-O and C-C, bond resonances, respectively. The peak of C-C bonds in C1s core-level spectrum relates to the sp^2^ hybridized carbon, represents carbon material, where as, C-O and C=O represent oxygen functionalities. O1s spectrum is divided into three different peaks at 533.0 eV (COOH groups), 532.1 eV (phenolic C-OH or C-O-C bonds) and 530.8 eV (quinonoid C=O bonds) as shown in Fig. 4b ^38^. These oxygen-functional groups are responsible for reversible pseudo-capacitive reactions.

In order to exposing the defects in the as prepared samples (RGOF, RHGOF and RHGOF/CNTs), Raman spectroscopy study is carried out, as shown in the Fig. 4d. The D band is ascribed to the sp^3^ hybridized carbon atoms and it reveals the defects is generated in the graphene basal plane^39^ and in graphitic materials first order scattering mode of sp^2^ hybridized carbon atoms within the lattice plane of the graphene materials correspond to G band^40^.

All three samples show different D and G band, (I_D_/I_G_) intensities ratios as shown in the Fig. 4d. From Fig. 4d, it is shown that I_D_/I_G_ ratio of RHGOF is increased as compared to RGOF,which is resulted from the formations of abundant pores and defects in the basal plane of graphene. Similarly remarkable increase of I_D_/I_G_ ratio for RHGOF/CNTs composite as compared to aforementioned composites elucidates that more defects is generated due to the CNT growth.

**Figure 5:**
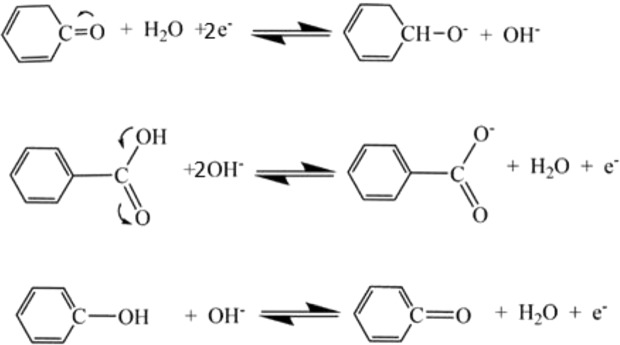
The various oxygen-functional groupsanticipated by reversible pseudo-capacitive reactions.

As shown in Fig. 5 the reversible redox conversions take place in quinonoid C=O bonds, phenolic C-OH bonds and COOH groups, which provided the pseudo-capacitance effect in the porous oxygen-doped carbon electrodes.

**Figure 6:**
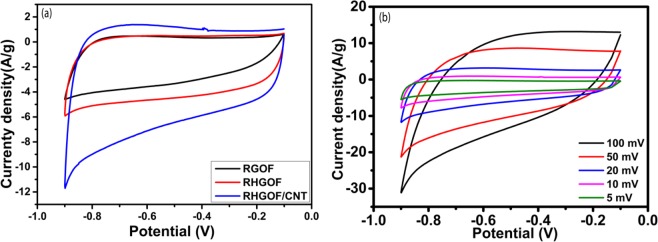
(**a**) CV curves of RGOF, RHGOF and RHGOF/CNTs at scan rate of 10 mV, (**b**) the CV curves of RHGOF/CNTs at different scan rates.

Capacitive properties of RGOF, RHGOF and RHGOF/CNTs hybrid electrodes were investigated in 6 M KOH aqueous electrolyte by CV, CD and EIS. CV curves of samples for RGOF, RHGOF, and RHGOF/CNTs are shown in Fig. 6a, measured between −0.1 V to −0.9 V potential windows at the scan rate of 10 mV/s. Figure 6b depicts, the CV curves of the RHGOF/CNTs hybrids at different scan rates.

The designed hybrid structure, during the scan rates ranging from 5 mV to 100 mV displays outstanding electrochemical behavior, which implies that 3D RHGOF/CNTs flexible electrode shows a good rate capability. Likewise, comparison of areas of CV curves between RHGOF electrode and RGOF electrode shows RHGOF has superior area than that of RGOF. It is also shown from the Fig. 6a that the CV curve area of the RHGOF/CNTs electrode is greater than that of both aforementioned electrodes. Consequently, due to contribution of the large integrated surface area, the specific capacitance is increased. In fact, due to the etching of graphene nano-sheets with a suitable amount of H_2_O_2_ during synthesis of RHGOF through hydrothermal process, the accessible specific surface area is increased. Also, it is shown that RHGOF/CNTs has largest accessible surface area as compared to the RGOF and RHGOF. It is evidenced that the CNTs grown within a sandwich structure act as a spacer, as confirmed by FESEM and TEM results. At the same scan rate, the improvement of cyclic voltammetry performance is attributed to the porosity of RHGOF as well as the higher accessible specific surface area.

**Figure 7:**
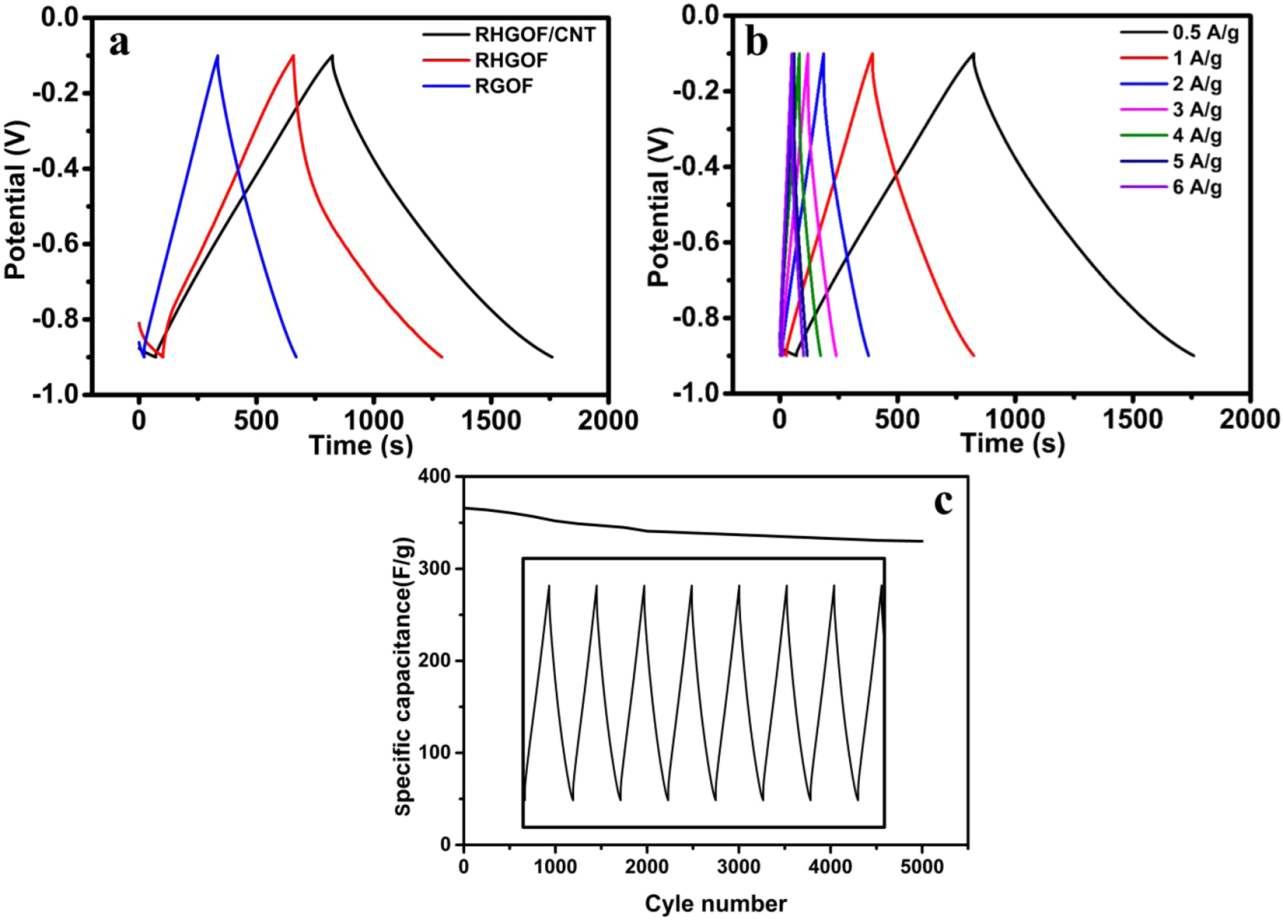
(**a**) CD curves of RGOF,RHGOF and RHGOF/CNTs at 0.5 A/g current density, (**b**) at different current densities CD curves of RHGOF/CNTs (**c**) Cyclic stablity of RHGOF/CNTs.

At different current densities the charge-discharge profile of RHGOF/CNTs shown in Fig. 7b. It is found that all electrodes possess nearly a symmetric charge-discharge curve of triangular shapes, illustrating that the electrical double layer capacitance (EDLC) is contributed main part in the specific capacitance. The faint deviation from the rectangular shape of CV curves and the charge-discharge profiles of electrodes display a complex supercapacitance, called pseudo-capacitance and EDLC. The major cause of pseudo capacitance is due to the oxygenated groups that are presented on the surface of electrodes. It is considered that the oxidization or reduction of electrochemically active oxygenated groups is responsible to slightly distorted CV curves contain faint redox peaks (in XPS analysis) in the electrolyte^41–43^. At current density 0.5 A/g, the specific capacitance of the synthesized electrodes are 557, 329 and 208 F/g for RHGOF/CNTs, RHGOF and RGOF respectively are shown in Fig. 7a. The RHGOF/CNTs exhibits longest charge-discharge profile among all the electrodes is attributed to the highest capacitance, which shows similar pattern as in the CV curves. Furthermore, RHGOF/CNTs composite shows greater value than those of reported similar electrodes such as self-assembled reduced graphene oxide/carbon nanotubes thin film has specific capacitance 428 F/g at 0.5 A/g^44^, GO/CNT sandwich paper has specific capacitance 151 F/g at 0.5 A/g^45^, holey GR paper (at 1 A/g, 201 F/g)^28^, GR/mesoporous carbon nanosphere film electrode (at 0.2 A/g, 211 F/g)^46^, multiwall carbon nanotubes/graphene film electrode (at 0.1 A/g, 265 F/g)^36^ and CNT/holey graphene hybrid film (at 0.25 A/g, 268 F/g)^33^, etc.

Figure 7a shows the charge discharge profile of as prepared samples at 0.5 A/g as the function of current density. The specific capacitance of RHGOF/CNTs is highest among all other electrodes.This highest capacitance can be attributed to the following considerations. At first, it is related to the porous network. A porous structure is supposed to produce pathways to electrolyte to penetrate deeply inside electrode and cut down the ion transport path. Hence, it significantly enhances the utilization of maximum area of RHGOF, as a result the conductive channel in the hybrid electrode is increased. Secondly, inside the RHGOF/CNT hybrid, CNTs acts as an electrically conductive connections, due to the diffusion of electrons and electrolyte ions inside the flexible electrode the average transport length of electrolyte ions is spectacularly shorten, hence charge-discharge process are facilitated during the reaction. Therefore, electrochemical as well as galvanostatic properties are enhanced. It is shown in Fig. 7c that the cyclic stability of RHGOF/CNTs is measured through the charge-discharge curve at 5 A/g. After 5000 consecutive charge discharge cycles, the capacitance retention RHGOF/CNTs is 90%.

**Figure 8:**
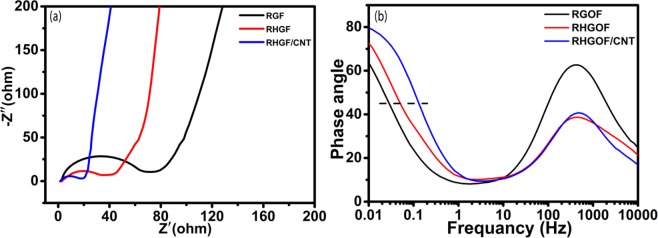
(**a**) EIS curves of RGOF, RHGOF and RHGOF/CNTs, (**b**) phase angle against frequency of RGOF, RHGOF and RHGOF/CNTscomposite.

Figure 8a shows Nyquist plot of RGOF, RHGOF and RHGOF/CNTs electrode at 5 mV signal voltage.

A close up view depicts that short 45^o^ Warburg region and semi circle for all three composites which are used as electrode. At high frequancy range equivalent series resistance (ESR) R_s_ is evaluated by real part of resistance (Z′) with real axis provided by ionic resistance of the electrolyte, contact resistance with current collector and the intrinsic resistance of the active material. Charge transfer resistance (R_ct_) at the interface between electrolyte and electrode is shown by middle-high frequency range semi circle region. This is also known as Faraday’s resistance. Figure 8a shows the semi circle of RGOF is largest than RHGOF and RHGOF/CNTs proving that RGOF has higher value of R_ct_ than other two electrodes. The span of semi circle of RHGOF is reduced than RGOF as shown in the Fig. 8a. Similarly, span of semi circle of RHGOF/CNTs is reduced than RHGOF is attributed to increasing number of conductive sites to transport charges,which is increased by porous structure generation and CNTs growth. The plot of RHGOF/CNTs electrode shows fast ion diffusion and ideal capacitive behavior among the three electrode denoted by the sharpest slope during low frequency range. Fast ion diffusion took place in RHGOF/CNTs electrode due to the highly porous micro structure. Hence it is evidenced that the electrolyte is directly contact with active material electrode. As a result the distance of ionic diffusion is decreased.

From the Fig. 8b it is shown that the dependence of impedance phase angle against the frequency for RGOF, RHGOF and RHGOF/CNT electrodes.All electrodes have discharge time with efficiency more than 50% is described as phase relaxation time constant(t_o_) of supercapacitor. We can find form the equation t_o_ = 1/ƒ_o_ at phase angle of −45° with same capacitive and resistive impedance^47^. The characteristic frequencies are measured to be 0.046, 0.082 and 0.1778 Hz at phase angle of 45° in 6 M KOH corresponding t_o_ of 21.73, 12.19 and 5.62 sec for RGOF, RHGOF and RHGOF/CNT, respectively. Smaller t_o_ of RHGOF/CNTs shows that higher accessible surface area was available to electrolyte ions to improving electrical conductivity as compared to RGOF, and RHGOF. It was also shown that t_o_ of RHGOF is smaller than RGOF,which evidences large accessible surface area that proved holes were present in the basal plane. In addition, due to very small relaxation time t_o_ of RHGOF/CNTs this conductive 3D structure is responsible for dynamics of ion diffusion of electrolyte in the inner of electrode.

## Conclusion

Sandwich RHGOF/CNTs hybrid material was successfully prepared by the combination of vacuum filtration and chemical vapor deposition. CNTs serve as conductive spacers in the RHGOF/CNTs structures, which prevent the restacking of RHGO layers. The 3D conductive interpenetrated architecture is perfect for fast ion penetration. The RHGOF/CNTs flexible composite is directly used as binder-free electrode. At 0.5 A/g, the current density of the RHGOF/CNTs hybrid reveals a high specific capacitance of 557 F/g. Moreover, the electrode displayed reasonable cycle stability and excellent rate capability.During the charge-discharge process this 3D structure is not only supportive to fast electron transfer but it is also beneficial in utilizing specific surface area to a greater extent. We thought that the flexible RHGOF/CNTs hybrid structure is a promising candidate for SECs electrodes.

## Experimental

### Synthesis of RHGOF, RGOF

Graphene oxide (GO) (XFNANO, Inc. Graphene oxide (GO) (XFNANO, Inc. Advance Material Supplier) was dispersed into ultra-pure water and treated under ultra-sonication for two hours (30 min sonication followed by 10 min stirring, consecutively) in water bath. Hence, a homogeneous suspension of GO (1 mg/ml) was obtained. Then, 0.4 ml of H_2_O_2_ (30%) was mixed into GO suspension (40 ml, 1 mg/mL) by continuous stirring for half an hour and we got a suspension. Afterwards 50 ml Teflon lined autoclave was used for hydrothermal reaction. Finally autoclave was placed inside the oven for hydrothermal reaction at 100 °C for 9 hours. Final product obtained was holey GO abbreviated HGO. To reduce the HGO, 0.67 ml hydrazine solution (50%) was poured into HGO suspension and after 5 min stirring as prepared HGO suspension is refluxed at 100 °C for one and half an hour and cooled down naturally to the room temperature. As a result, after reduction HGO suspension changed into reduced holey graphene oxide solution and final solution was named as RHGO. Finally, reduced holey graphene oxide film (RHGOF) was prepared via vacuum filtration by using filter paper with pore size of 0.45 µm. As an experimental comparison, reduced graphene oxide film (RGOF) was synthesized by using the same conditions to prepare RHGOF, without adding H_2_O_2_.

### Preparation of RHGOF/CNTs sandwich structure

CNTs were grown using the ferric salt (FeCl_3_) as a precursor in formation of catalytic iron nano particles. 0.0135145 g of FeCl_3_ was dissolved in 50 ml deionize water to prepared 0.001 M catalytic solution. Afterwards, RHGOF was kept dipping in the prepared solution for 12 hours so that iron catalytic solution penetrates into the RHGOF. Than as prepared sample was dried at room temperature for 12 hours naturally. The CNTs were grown by using chemical vapor deposition (CVD) methods while C_2_H_2_ was used as carbon source. Catalyst carrying sample was placed into a quartz tube on a Si strip, and transferred into the center of CVD chamber. First the as prepared sample was heated at 710 °C for 9 min, while Ar (470 sccm) atmosphere is provided before supplying carbon source. Afterwards a mixture of C_2_H_2_ (18 sccm) and Ar (457 sccm) was simultaneously introduced into the chamber for 5 min to grow CNTs. Finally, the sample was naturally cooled down to room temperature under the Ar atmosphere with a flow rate of 470 sccm.

### Characterization of as prepared samples

The structural characterization of as prepared samples were characterized by a field emission scanning electron microscopy (QUANTA 450, 20 KV,FESEM), moreover morphology was also studied by using high-resolution transmission electron microscopy (JEOLJEM-200,HRTEM). The defects in the prepared samples were studied by using a Raman spectroscopy (Renishawin Via Plus, He-Ne laser, 532 nm, 50% laser power). The atomic composition and surface chemistry of as prepared sample was studied by an X-ray photo electron spectroscopy (Al-K X-ray source, XPS, ESCALAB 250). The pore size, accessible surface area and at relative pressure (P_o_/P) adsorption data in the range of 0.05–0.20 of the prepared sample was measured by using Brunauer-Emmett-Teller(BET) method. Mechanical strength was measured by using universal material testing machine (Yl-S370) with crosshead speed 1 mm/min.

## Supplementary information

Supplementary information.
