# Supplementary material for: Reduced holey graphene oxide film and carbon nanotubes sandwich structure as a binder-free electrode material for supercapcitor
Source: Sci Rep. 2020 Feb 11;10:2315. doi: 10.1038/s41598-020-58162-9 (PMC7012873; doi:10.1038/s41598-020-58162-9)
Supplement: Supplementary file 1 — Supplementary information. [file 41598_2020_58162_MOESM1_ESM.docx]

**Supplementary information for**

**Reduced holey graphene oxide film and carbon nanotubes sandwich structure as a binder-free electrode material forsupercapcitor**

Khan Abdul Sammed^a^, Lujun Pan^a*, 1^,Muhammad Asif^b^,MuhammadUsman^a^,Tianze Cong^a^,FaridAmjad^a^,Muhammad Asif Imran^c^

^a^ School of Physics and Optoelectronic Technology, Dalian University of Technology, Dalian 116024, PR of China

^b^Department of Materials Science and Engineering College of Engineering ,Peking University,Beijing100871,China

^c^ School of Chemical Engineering, Dalian University of Technology, Dalian 116024, PR of China



 *Corresponding Author: Tel: 86-411-84707863-334; Fax: 86-411-84709304Email: lpan@dlut.edu.cn (Lujun Pan)


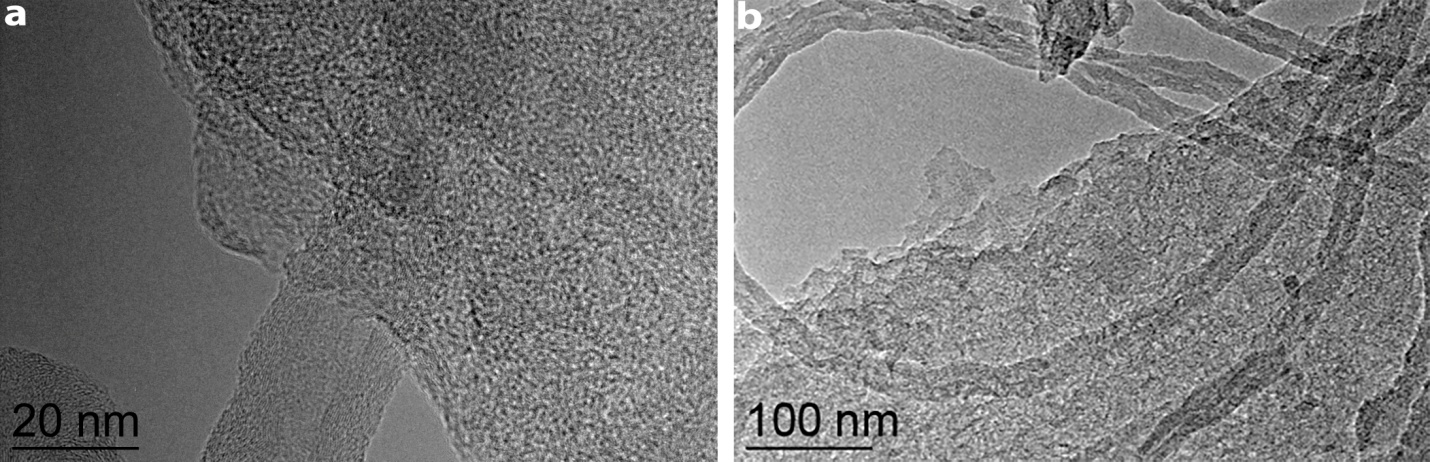


**Fig.(s1)** HRTEM image of RHGOF/CNTs hybrid composite at different resolution


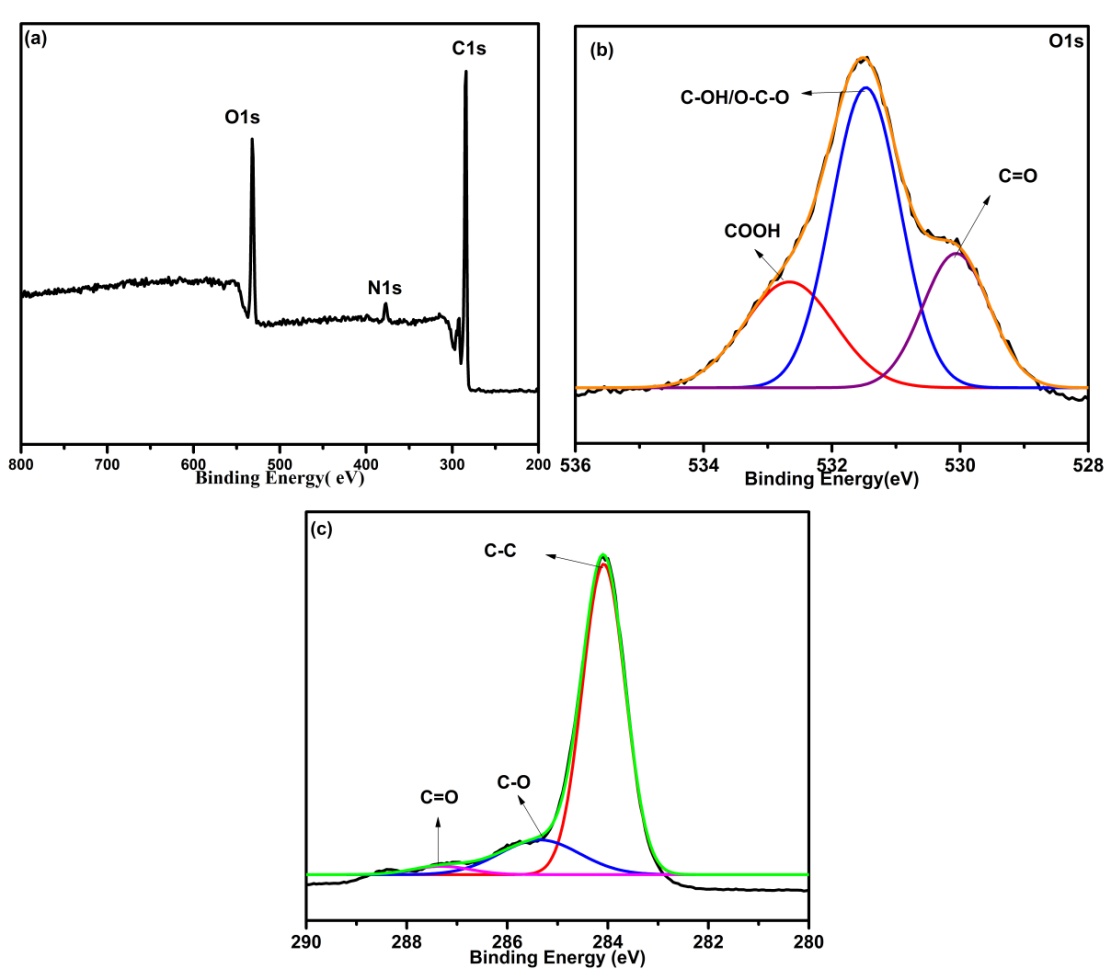


**Fig.(s2)** (a) XPS survey, (b) O1s spectra, and (c) C1s spectra for RGOF


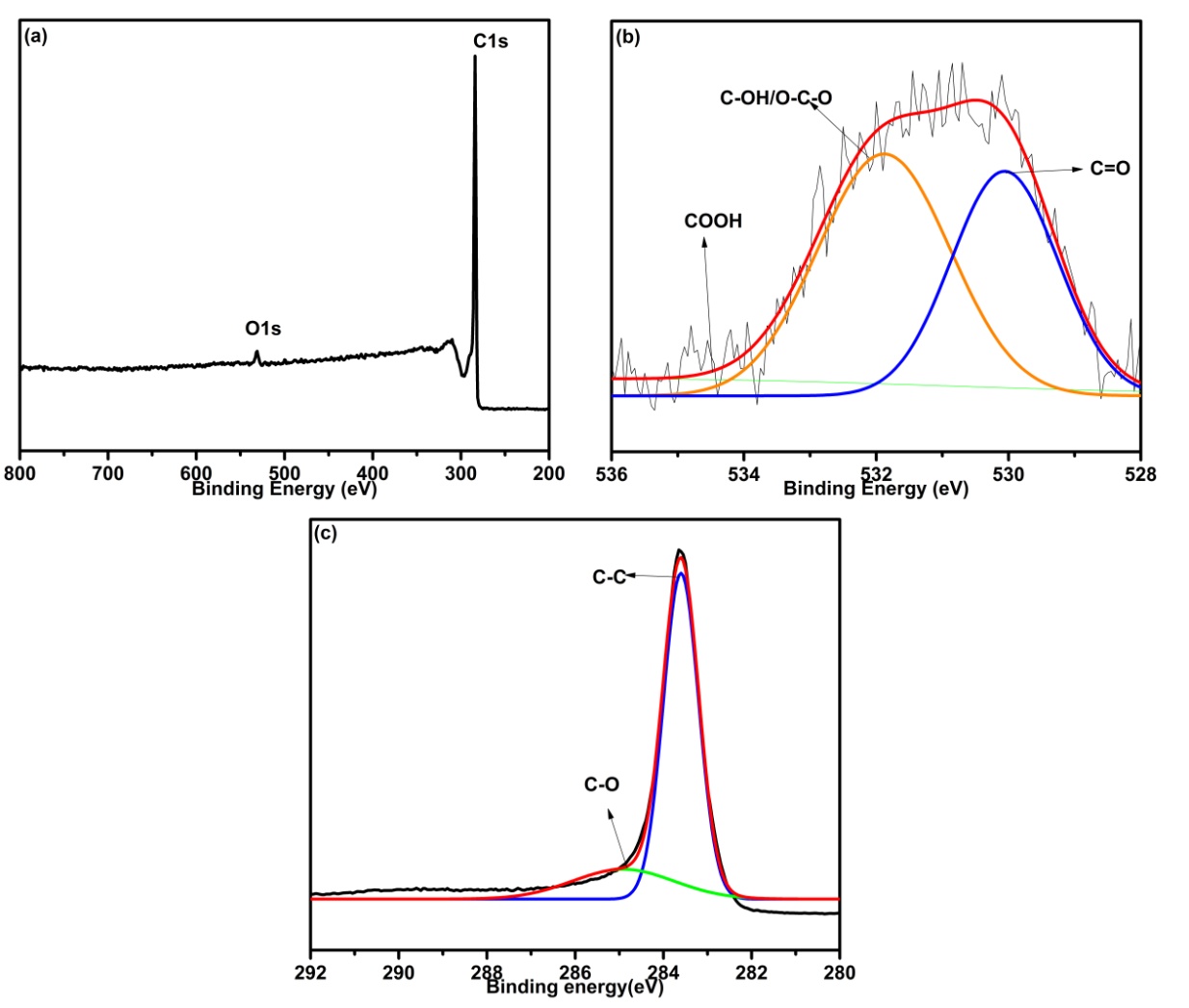


**Fig.(s3)** (a) XPS survey, (b) O1s spectra, and (c) C1s spectra for RHGOF/CNTs

Surface Area Data

Multipoint BET 2.546e+02 m²/g

BJH method cumulative adsorption surface area 5.911e+02 m²/g

BJH method cumulative desorption surface area 1.620e+03 m²/g

DH method cumulative adsorption surface area 6.340e+02 m²/g

DH method cumulative desorption surface area 1.834e+03 m²/g

**Surface area detail of RGOF**

Surface Area Data

Multipoint BET 5.608e+02 m²/g

BJH method cumulative adsorption surface area 5.503e+02 m²/g

BJH method cumulative desorption surface area 6.932e+02 m²/g

DH method cumulative adsorption surface area 5.760e+02 m²/g

DH method cumulative desorption surface area 7.320e+02 m²/g

**Surface area detail of RHGOF**
